# The Spleen Plays No Role in Nephrotoxic Serum Nephritis, but Constitutes a Place of Compensatory Haematopoiesis

**DOI:** 10.1371/journal.pone.0135087

**Published:** 2015-08-06

**Authors:** Katharina Artinger, Alexander H. Kirsch, Ida Aringer, Corinna Schabhüttl, Alexander R. Rosenkranz, Philipp Eller, Elena Rho, Kathrin Eller

**Affiliations:** 1 Clinical Division of Nephrology, Department of Internal Medicine, Medical University of Graz, Graz, Austria; 2 Intensive Care Unit, Department of Internal Medicine, Medical University of Graz, Graz, Austria; 3 Clinical Division of Internal Medicine, Ospedale La Carità, Locarno, Switzerland; Wayne State University, UNITED STATES

## Abstract

**Background:**

The spleen has been implicated in the pathogenesis of immune-complex glomerulonephritis by initiating and resolving adaptive immune responses. Thus, we aimed to evaluate the role of the spleen in experimental nephrotoxic serum nephritis (NTS).

**Methods:**

In order to accelerate the disease, animals were subjected to NTS by preimmunizing male C57BL/6J mice with rabbit IgG three days before injecting the rabbit anti-glomerular basement antiserum, or were immunized only. A group underwent splenectomy before NTS induction.

**Results:**

We observed enlargement of the spleen with a maximum at 14 days after NTS induction or immunization only. Splenectomized mice were found to develop albuminuria and renal histological changes comparable to sham-operated controls. Nevertheless, anaemia was aggravated in mice after splenectomy. During the course of NTS, we detected CD41^+^ megakaryocytes and Ter119^+^ erythroid precursor cells in the spleen of mice with NTS and of immunized mice. Ter119^+^Cxcr4^+^ cells and the binding partner Cxcl12 increased in the spleen, and decreased in the bone marrow. This was accompanied by a significant systemic increase of interferon-gamma in the serum.

**Conclusions:**

In summary, splenectomy does not influence the course of NTS *per se*, but is involved in concomitant anaemia. Extramedullary haematopoiesis in the spleen is probably facilitated through the migration of Cxcr4^+^ erythroid precursor cells from the bone marrow to the spleen via a Cxcl12 gradient and likely arises from the suppressive capacity of chronic inflammation on the bone marrow.

## Introduction

Glomerulonephritis (GN) is still an important cause of end-stage renal disease in a significant number of patients needing renal replacement therapy. Even though research within the past decades led to a better understanding of the pathogenesis and to new therapeutic options in GN, the disease itself and current standard therapy are still accompanied by a reduction in life expectancy and quality. Experimental nephrotoxic serum nephritis (NTS) is a murine model of immune-complex GN closely resembling forms of human rapid progressive GN [1–3]. This rapidly progressive disease is induced by the injection of rabbit anti-mouse glomerular basement membrane (GBM) antibody and accelerated by a preceding immunization against rabbit IgG. Animals with NTS present with proteinuria and proliferative and inflammatory glomerular changes, including crescent formation and kidney infiltrating leukocytes [2]. The pathogenesis depends on T helper cells type 1 and type 17 as well as regulatory T cells [1,2,4–6]. Previous research of our group found compelling evidence that secondary lymphoid organs such as the draining lymph nodes and the spleen play an important role in the pathogenesis of NTS, since various leukocytes, including regulatory T cells, home to these organs [1,2,7]. Furthermore, the spleen is involved in antibody production, clearance of immune complexes as well as antigen-presentation, all of which are key factors in the development of NTS. Nevertheless, the functional role of the spleen in NTS is unknown so far.

In human GN data on the functional role of the spleen are scarce. Case reports provided anecdotal evidence of a potential protective, but also harmful effect of splenectomy in patients with GN due to cryoglobulinaemia, IgA nephritis and connective tissue disease [8–10].

Anaemia in GN develops on the one hand due to the lack of erythropoietin production in the kidney and on the other hand due to the chronic inflammation [11,12]. There are currently no published data on compensatory mechanisms of possibly accompanying anaemia in the NTS model.

Here, we provide evidence that splenectomy does not influence the course of NTS, but aggravates concomitant anaemia. After the induction of NTS, mice develop bone marrow depression and extramedullary splenic haematopoiesis in an environment shaped by systemic inflammation. Our data point towards a migration of Cxcr4^+^ erythroid precursor cells from the bone marrow to the spleen via a Cxcl12 gradient.

## Material and Methods

### Ethics statement

All animal experiments were approved by the Committee on the Ethics of Animal Experiments of the Austrian Ministry (BMWF-66.010/o121-II/3b/2014). All animal experiments were conducted under strict adherence to the laws of Austria (BGBl. I Nr. 118/2004). All efforts were made to minimize suffering.

### Induction of NTS and mice

C57BL/6J mice were either obtained from Charles River Laboratories (Sulzfeld, Germany) or Himberg (Vienna, Austria). All experiments were performed in 8- to 12-week-old, male mice, maintained in a virus-free environment. NTS was induced as described previously [3]. Briefly, 3 days before administration of the nephrotoxic serum, mice were subcutaneously preimmunized with 2 mg/ml rabbit IgG (Jackson ImmunoResearch Laboratories, West Grove, PA, USA) dissolved in incomplete Freund´s adjuvant (Sigma-Aldrich, St. Louis, MO, USA) with desiccated, nonviable Mycobacterium tuberculosis H37a (Difco Laboratories, Detroit, MI, USA). Heat-inactivated rabbit anti-mouse glomerular basement membrane serum was prepared as described previously [13] and injected three days after immunization intravenously via the tail vein.

### Splenectomy and study design

Eight-to 12-week old male C57BL/6J mice were anesthetized using 6–8 mg/kg bodyweight xylazine and 90–120 mg/kg bodyweight ketamine and a sickle-shaped incision was made on the central abdomen. Carefully avoiding bowels and intestines, the spleen was exposed. After preparation of the vascular bundles, two ligations were placed on each bundle in order to remove the spleen. Sham-operated mice underwent the same procedure without removal of the spleen. After a two-week period of recovery, NTS was induced and mice were either killed after 14 or 28 days of NTS. Moreover, mice were subjected to NTS or immunization only and followed until day 7 or 14 without preceding sham-operation or splenectomy.

### Urinary albumin and urinary creatinine detection

A double-sandwich ELISA (Abcam, Cambridge, MA, USA) was used for determination of urinary albumin excretion as reported previously [2]. Urinary creatinine was evaluated photometrically using a commercially available picric acid-based kit (Sigma-Aldrich).

### Evaluation of histopathology

For Periodic-Acid-Schiff (PAS)—and Haematoxylin-Eosin (HE)-stainings, formalin-fixed renal and spleen tissue was embedded in paraffin and cut in 4 μm sections prior to staining. For evaluation of PAS-stainings, a minimum of 50 glomerular cross-sections were evaluated as described previously [14]. Shortly, PAS^+^ material was scored within glomeruli following a semiquantitative scoring system consecutively numbered 0–3.

### Evaluation of immunopathology

Frozen tissue sections (4μm) were cut for immunoperoxidase stainings and immunofluorescence. A three-layered immunoperoxidase staining technique was used for the assessment of tissue infiltrating or resident cells. Kidney sections were stained using rat-derived primary antibodies for CD4 (clone YTS191.1; Serotec, Oxford, UK), CD8 (clone KT15, Serotec), CD68 (clone FA-11; Serotec) and an anti-Neutrophil antibody (clone NIMP-R14; Abcam). Spleens were stained with primary antibodies for F4/80 (clone CI:A3-1; Serotec) and CD41 (clone MWReg30; BD Pharmingen, San Diego, CA, USA). A biotin-conjugated goat anti-rat IgG (Jackson ImmunoResearch Laboratories) was used as a secondary antibody.

While cell numbers of infiltrating CD4^+^, CD8^+^ T cells and neutrophils were counted in 6 high-power fields of renal cortex and medulla, infiltrating macrophages were evaluated using a semiquantitative scoring system as listed below: 0 = 0 to 4 cells stained positive, 1+ = 5 to 10 cells, 2+ = 11 to 50 cells, 3+ = 51 to 200 cells, and 4+ >200 cells stained positive per low-power field. Evaluation of F4/80 positive cells, which are indicative for the red pulp, was done using Aperio ImageScope software 11.1.2.760 (Leica Biosystems, Nussloch, Germany) and the positive pixel count algorithm (hue value: 0.1; hue width: 0.5; color saturation threshold 0.04). Scanning of slides was performed with Aperio ScanScope AT (Leica Biosystems).

For direct immunofluorescence of autologous IgG, FITC-conjugated goat anti-mouse IgG (Jackson ImmunoResearch Laboratories) in serial dilutions and for erythroid progenitor cells FITC-conjugated antibody for Ter119^+^cells (clone TER-119; eBioscience, San Diego, CA) was used and eventually mounted with mounting medium for fluorescence with DAPI (Vector Laboratories, Burlingame, CA, USA).

### Assessment of autologous and heterologous antibody responses

After overnight incubation with 100 μg/ml rabbit IgG (Jackson ImmunoResearch Laboratories), serum from peripheral blood was incubated with serial-doubling dilutions for the detection of circulating mouse anti-rabbit immunoglobulin. Using HRP-conjugated goat anti-mouse IgG and goat anti-mouse IgG1, IgG2b and IgG3 (all from Jackson ImmunoResearch Laboratories), mouse anti-rabbit IgG and respective subclasses were detected. For the assessment of circulating rabbit immunoglobulin antibodies, a HRP-conjugated goat anti-rabbit IgG (Jackson ImmunoResearch Laboratories) was used.

### Assessment of serum IFN-γ, Il-6 and TNF-α

IFN-γ, Il-6, and TNF-α levels in serum were determined using commercially available ELISA kits (all from BD, San Jose, CA, USA).

### Reverse transcription (RT) real-time polymerase chain reaction (PCR)

Total RNA was extracted from spleens and kidneys using TRI Reagent (Sigma-Aldrich) and subsequently cDNA was synthesized by using Superscript III Transcription Kit (Invitrogen, Carlsbad, CA, USA) and random primers (Invitrogen) for reverse transcription of 2 μg of total RNA. Real-time PCR was performed in duplicates on a CFX96 Real-Time System (BioRad, Hercules, CA, USA).

For quantification of chemokines, we used TaqMan gene expression assays (Applied Biosystems, Foster City, DA, USA) for *Cxcl12* (Mm00445553_m1), *Ccl2* (Mm00441242_m1) and *Ccl7* (Mm00443113_m1). *18S RNA* (Mm03928990_g1) was used as a reference for spleen samples, while *Hprt* served as a reference gene for bone marrow samples and was assessed using SYBR Green Mastermix (Invitrogen) with the following primers: forward 5´GCT TCC TCC TCA GAC CGG TTT TTG C 3´; reverse 5´ATC GCT AAT CAC GAC GCT GGG ACT G 3´.

### Flow Cytometry

Cell suspensions from spleens and bone marrow were stained with APC-conjugated anti-human/mouse CD44 (clone IM7; eBioscience), FITC-conjugated anti-mouse Ter119 (clone TER-119; eBioscience) and eFluor450-conjugated anti-mouse CD184 (Cxcr4) (clone 2B11, eBioscience). In order to stain spleen- and lymph node-populations, the following antibodies were used: APC-conjugated rat anti-mouse CD4 (Clone RM4-5; BD Biosciences, San Jose, CA, USA), FITC-conjugated anti-mouse CD8a (clone 53–6.7; BioLegend, San Diego, CA, USA), PE-conjugated anti-mouse CD69 (clone H1.2F3; BioLegend) PE-conjugated anti-mouse B220 (clone RA3-6B2; BioLegend) and eFluor450-conjugated anti-mouse CD11b (clone M1/70; eBioscience). Samples were analysed on LSRII and FACSCalibur cytometers (both BD Biosciences).

### Statistical analyses

All statistical evaluations were performed using GraphPad Prism 6.0 for Macintosh (GraphPad Software, La Jolla, CA, USA) and results are shown as means ± SEM. Testing for normality was done using the Kolmogorov-Smirnov test with Dallal-Wilkinson-Lillifors correction and the Shapiro-Wilk normality test.

When comparing two groups, according to the distribution an unpaired t-test was used. A two-tailed p<0.05 was considered statistically significant. When comparing three or more groups, ANOVA or Kruskal-Wallis test was performed. In case of detected significance, two tailed t-tests or the nonparametric Mann-Whitney U test were performed and the Bonferroni method was used to adjust for multiple testing.

## Results

### NTS and immunisation cause a change in spleen morphology

**Fig 1:**
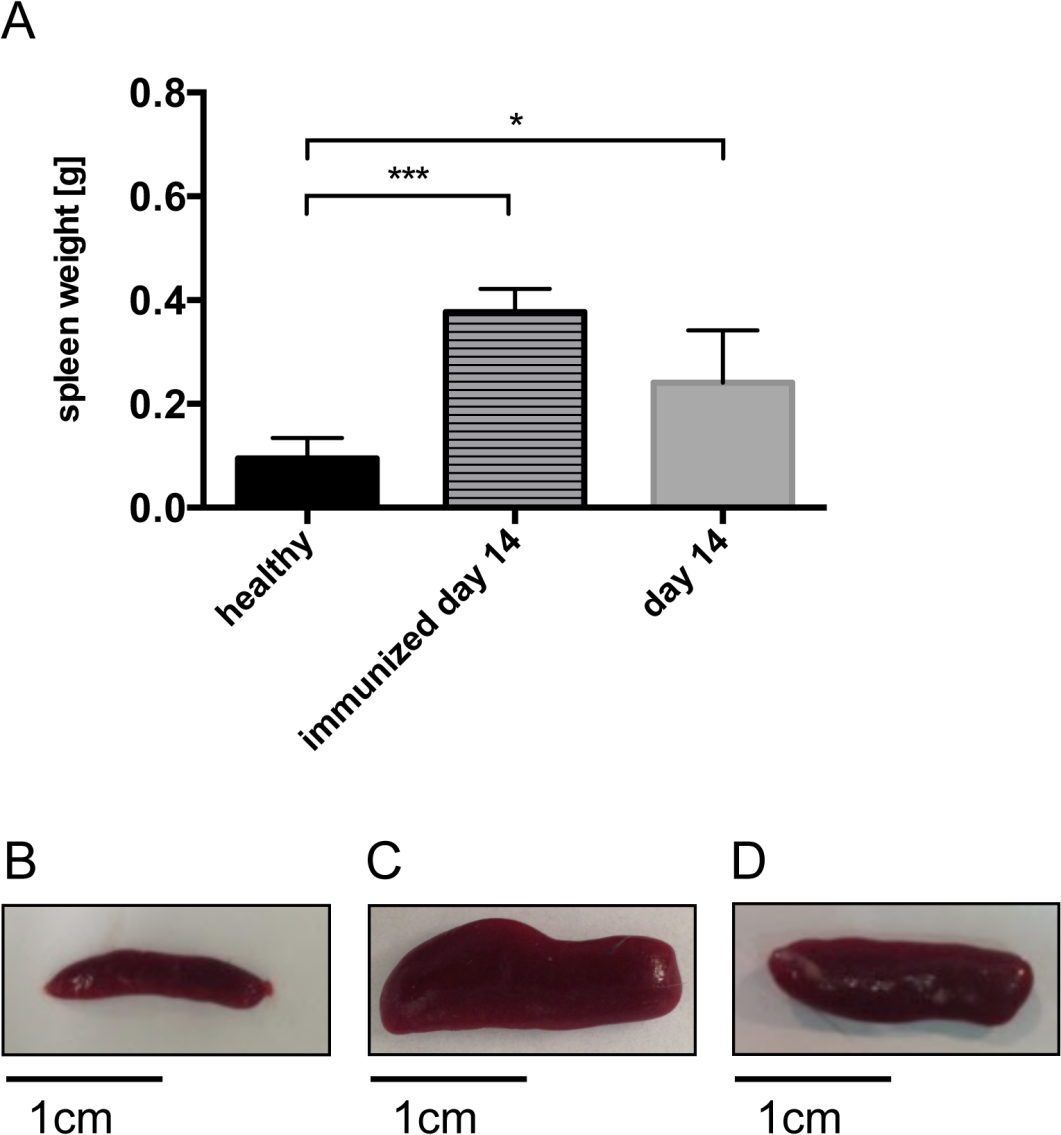
Mice with NTS and immunized mice gradually show increases in spleen size and weight. (A) Mean spleen weight is increased in immunized mice and in NTS on day 14. Spleen weight is given from healthy mice (black bar, n = 7), immunized mice (lined bar, n = 5), and mice with NTS on day 14 (grey bar, n = 7). (B, C, D) Representative pictures of spleens from a healthy mouse (B), an immunized mouse (C) and a mouse with NTS on day 14 (D) are shown. Data are given as means ± SEM.*p<0.05; ***p<0.001.

The role of the spleen in NTS has not been elucidated so far, even though a significant increase in spleen weight is observed 14 days after induction of NTS (Fig 1 and S1 Table). Of note, this was also true for mice, which were only immunized and did not receive the nephrotoxic antiserum (Fig 1).

### Splenectomy does not influence the phenotype of NTS

**Fig 2:**
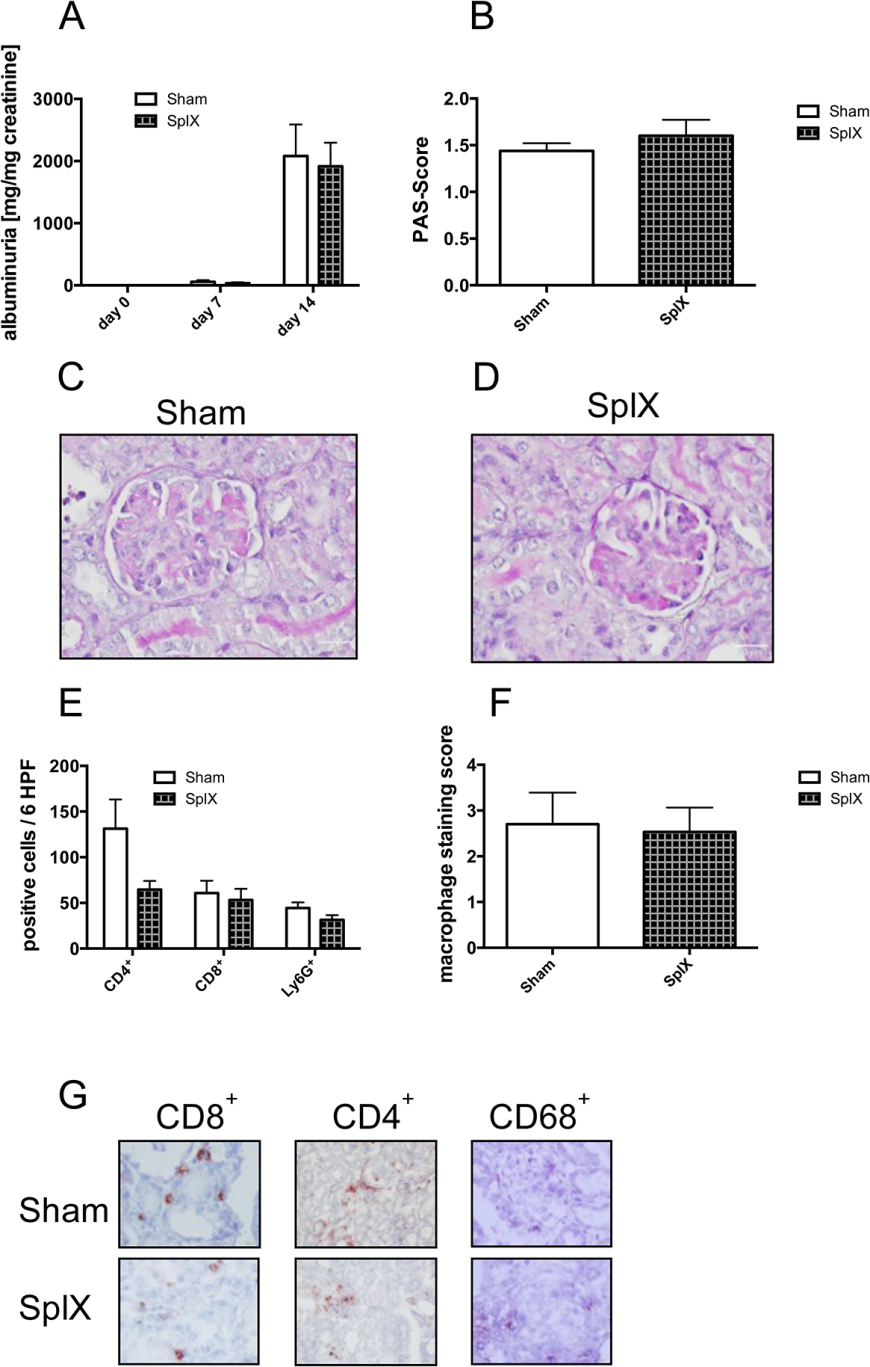
Splenectomy does not alleviate the kidney phenotype in NTS. Subsequently to either sham-operation (Sham, white bars, n = 5) or splenectomy (SplX, squared bars, n = 5), NTS was induced and albuminuria (A) on respective time points and PAS-Scores (B) on day 14 were evaluated. Representative PAS-stained kidney sections of a sham-operated (C) and a splenectomised (D) mouse are shown. Kidney infiltrating CD4^+^ and CD8^+^ T cells (E) as well as Ly6G^+^ neutrophil granulocytes (E) and CD68^+^ macrophages (F) were evaluated by performing immunohistochemical stainings from kidneys of sham-operated and splenectomized mice. Representative immunohistochemical stainings for CD4^+^, CD8^+^ cells and CD68^+^ macrophages are shown (G). Data are given as means ± SEM and are representative of three independent experiments with at least n = 4 in each group. Magnification x1000 (C, D, G) except for CD68^+^ (G), where magnification is x600.

Therefore, we performed splenectomy or sham operation in C57BL/6J mice and subjected them to our NTS model after a postoperative recovery phase of 2 weeks. There was no significant difference in albuminuria 7 and 14 days after NTS induction (Fig 2A and S2 Table). Furthermore, we detected no differences in PAS positive deposits in glomeruli as a marker of glomerular damage (Fig 2B and S3 Table). In line, crescent formation and tubular casts were equal in both groups (data not shown). Overall, kidney pathology after NTS induction was not altered by splenectomy (Fig 2C and 2D). Additionally, we found no difference in the amount of infiltrating cells such as CD4^+^ and CD8^+^ T cells, Ly6G^+^ neutrophils as well as CD68^+^ macrophages in kidneys of splenectomized or sham operated mice 14 days after NTS induction (Fig 2E–2G and S4 Table). Previously we provided compelling evidence that immune regulation takes place in the regional lymph nodes [1,2,15]. Thus, we performed flow cytometry for lymphatic T cell subpopulations from the draining lymph nodes, but no differences between the sham-operated and splenectomized group were detectable (data not shown).

### The B cell response is not altered by splenectomy in NTS

**Fig 3:**
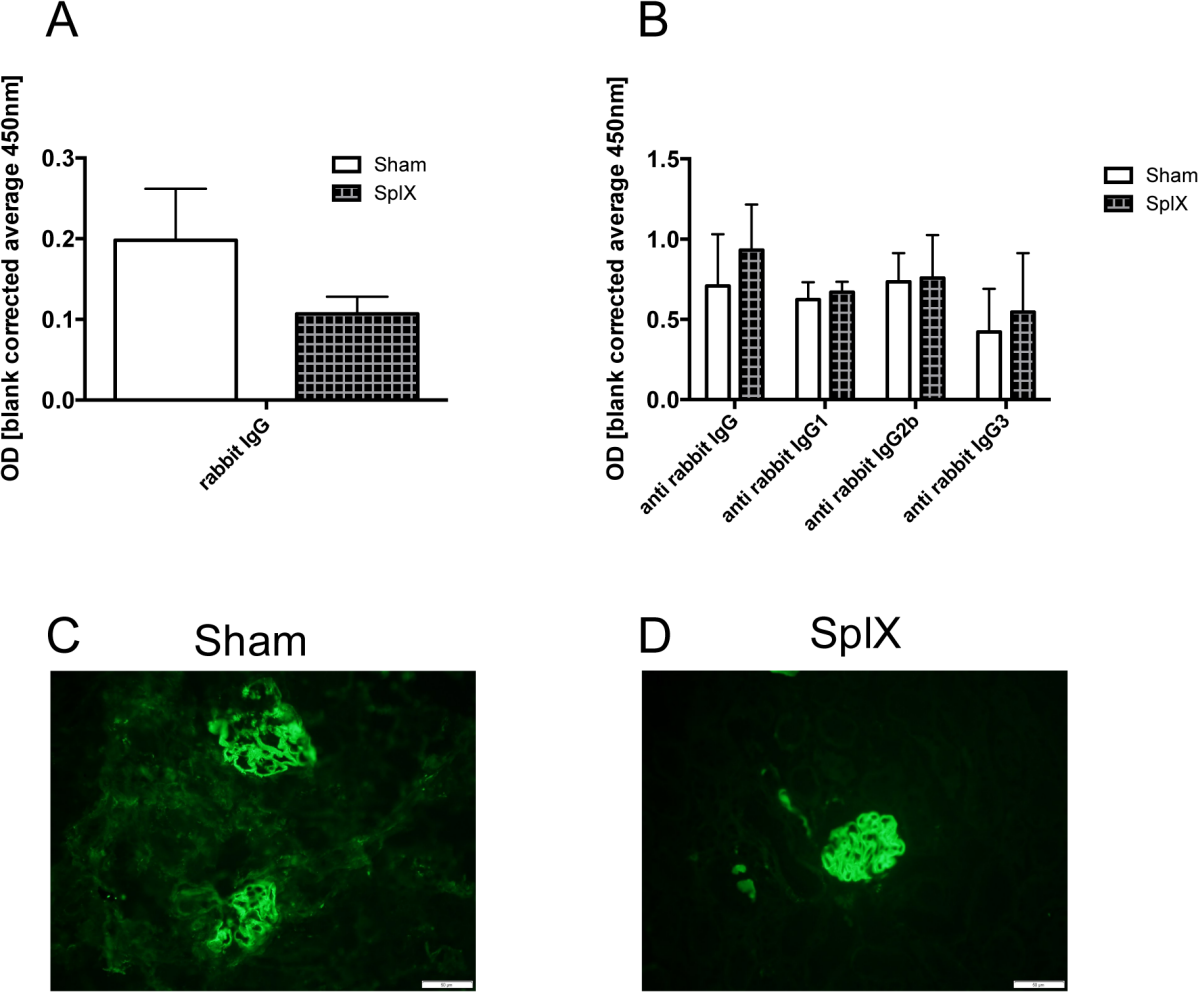
Splenectomy does not alter the presence of heterologous and autologous IgG in serum and kidneys. On day 14 serum of sham-operated (white bars, n = 5) and splenectomized mice (squared bars, n = 5) was evaluated for circulating rabbit IgG (A) and mouse anti-rabbit IgG as well as IgG subclasses (B). Data are given as means ± SEM and are representative of three independent experiments with at least n = 4 in each group. Evaluation of deposited autologous mouse anti-rabbit IgG was performed by immunofluorescent stainings from kidney sections for mouse anti-rabbit IgG 14 days after NTS induction. Two representative pictures of glomeruli from a sham-operated (C) and a splenectomized (D) mouse are shown. Magnification x200.

Interestingly, rabbit IgG (Fig 3A and S5 Table) as well as mouse anti-rabbit IgG and IgG subclass titre (Fig 3B and S5 Table) were identical in the serum of splenectomized and sham operated mice 14 days after NTS induction. Moreover, deposition of mouse anti-rabbit IgG (Fig 3C and 3D) and rabbit IgG (data not shown) in glomeruli 14 days after NTS induction did not differ between both groups. Of note, we also did not detect differences in the renal phenotype when mice were followed until day 28 after NTS induction (data not shown).

### Splenectomy aggravates anaemia after NTS

**Fig 4:**
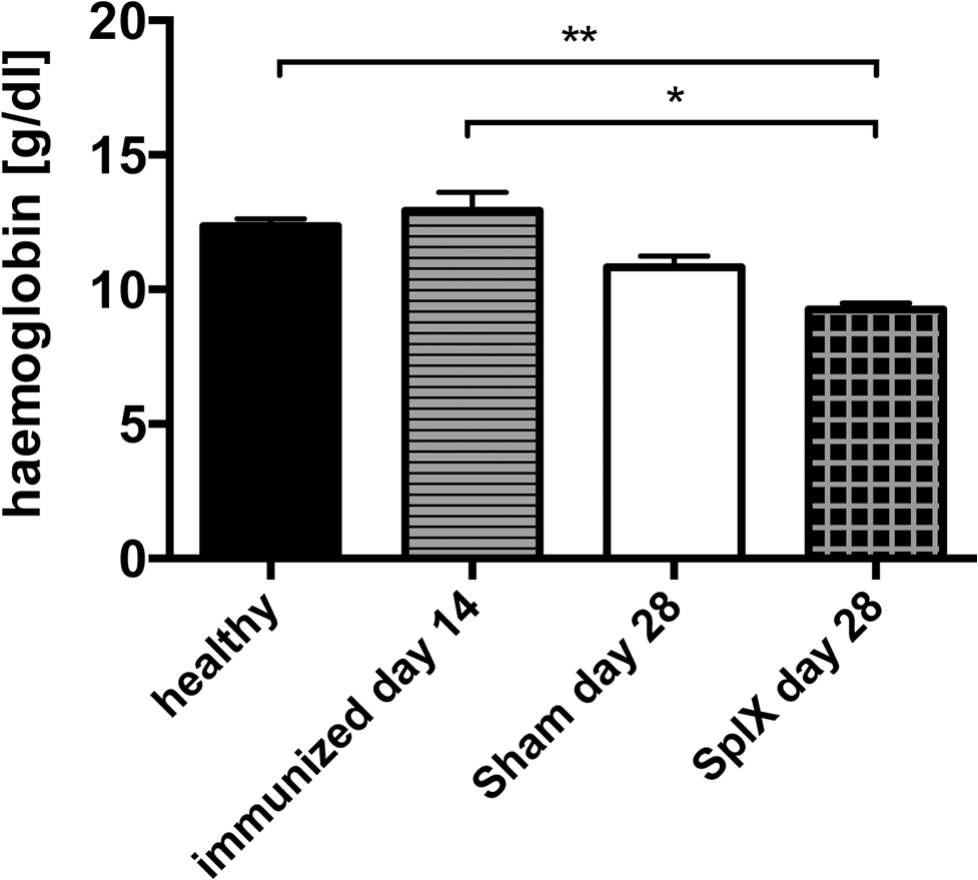
Splenectomy aggravates anaemia in NTS. Haemoglobin levels of healthy mice (black bar, n = 4) and immunized mice (lined bar, n = 4), as well as haemoglobin levels of sham-operated mice on day 28 (white bar, n = 4), and splenectomized mice with NTS (squared bars, n = 4) on day 28 were analysed. All data are given as means ± SEM. *p<0.05; **p<0.01.

Haemoglobin levels decreased until day 28 of NTS (Fig 4 and S6 Table). Even though there was no difference in the kidney phenotype of NTS, we observed significantly decreased haemoglobin levels in splenectomized mice 28 days after NTS as compared to healthy and immunized only mice (Fig 4).

### Spleen weight increases during NTS due to extramedullary haematopoiesis

**Fig 5:**
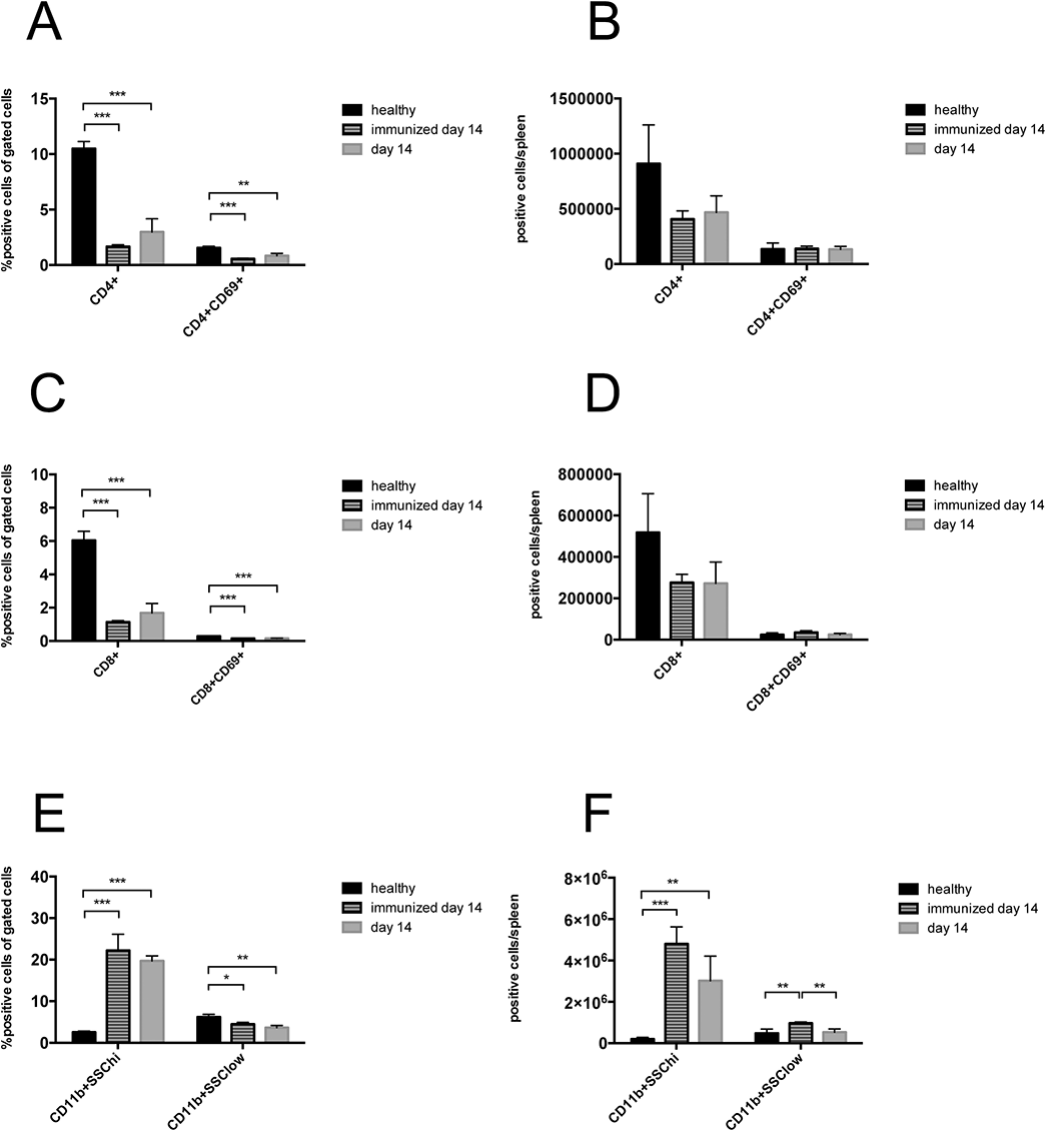
Enlargement and increase in spleen weight in immunized mice and in NTS is not attributable to leukocytes. CD4^+^CD69^+^ T cells (A, B) and CD8^+^CD69^+^ T cells (C, D) were analysed in spleens of healthy mice (black bars, n = 4), immunized mice on day 14 (lined bars, n = 4) and of mice 14 days (grey bars, n = 4) after NTS induction (A, B) by relative (A, C) and quantitative (B, D) flow cytometry. (E, F) Monocytes (CD11b^+^SSC^low^) and neutrophil granulocytes (CD11b^+^SSC^hi^) were evaluated relatively (E) and quantitatively (F) in spleens of healthy (n = 4), immunized (n = 4) and nephritic mice on day 14 (n = 4). All data are given as means ± SEM. *p<0.05; **p<0.01; ***p<0.001.

**Fig 6:**
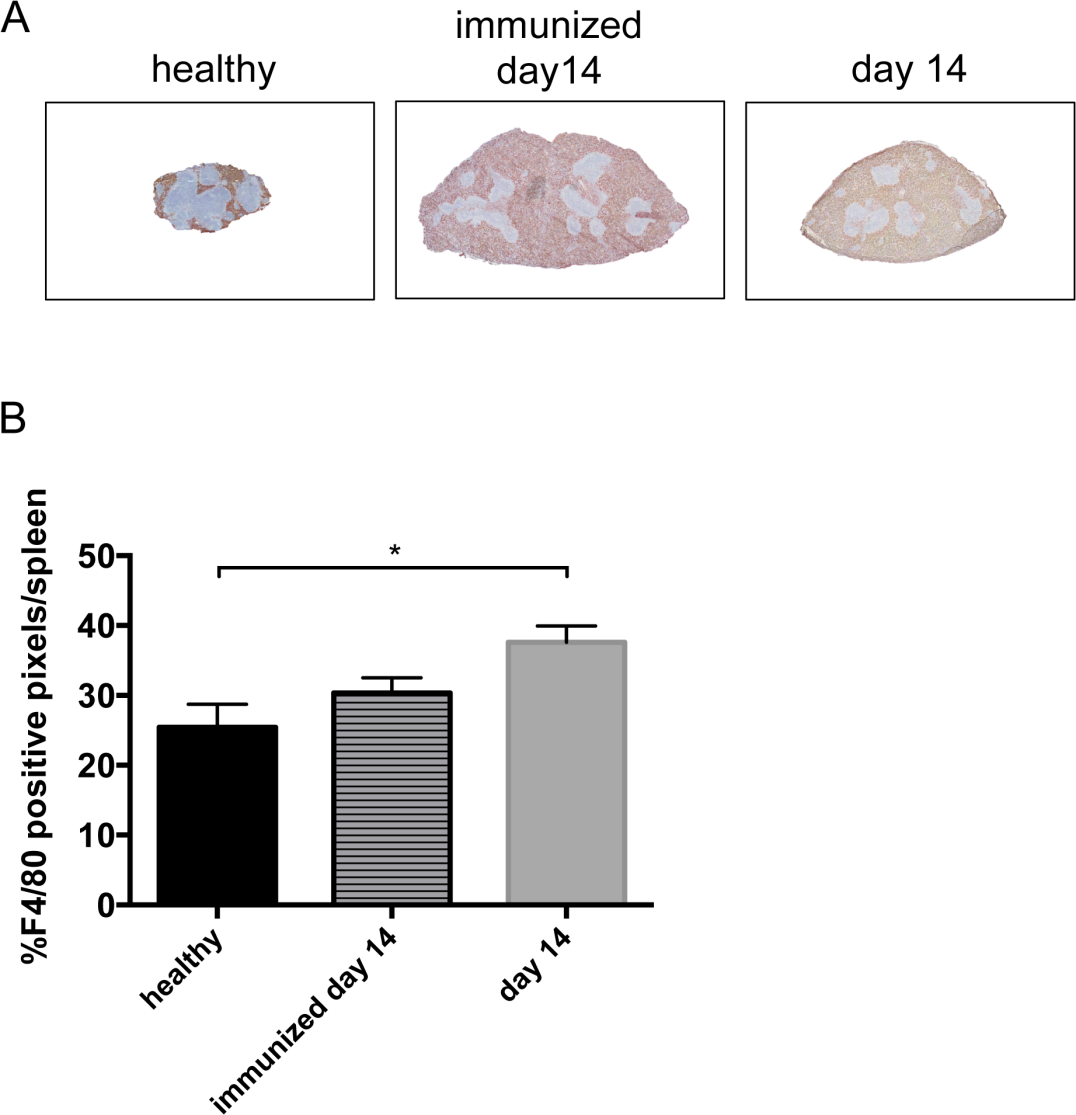
The red pulp increases in NTS. (A) Spleens from healthy (black bar, n = 5), immunized (lined bar, n = 5) and nephritic mice (grey bar, n = 5) on day 14 were stained for F4/80, indicating the red pulp. Representative pictures are shown. Magnification x40. (B) Red and white pulp were quantified for F4/80 positive cells. Data are given as means ± SEM. *p<0.05.

**Fig 7:**
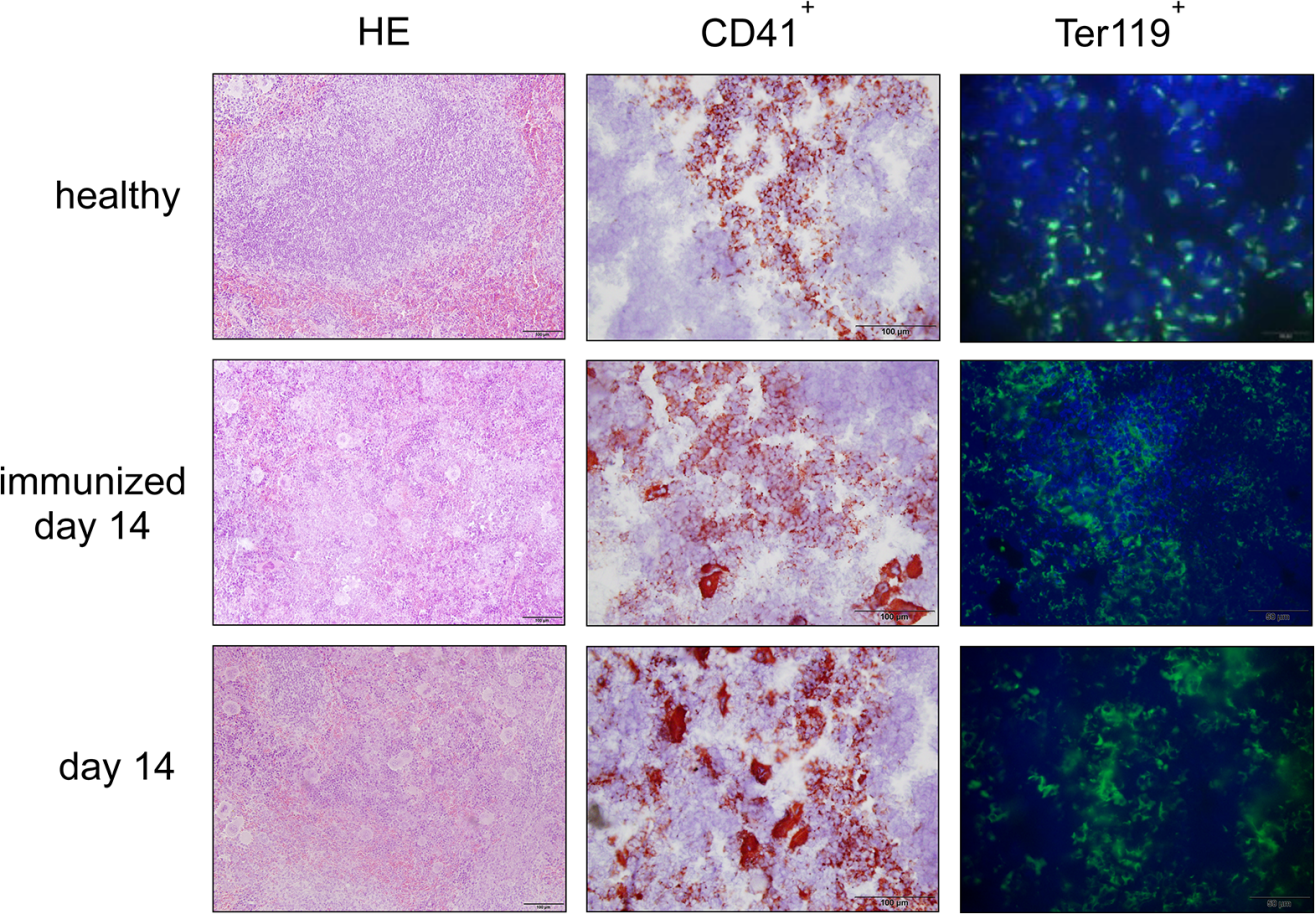
Extramedullary haematopoiesis is observed in spleens of immunized mice and 14 days after NTS induction. HE stains and immunohistochemical stainings for CD41 and Ter119 were performed from spleens of healthy mice, immunized mice, and mice with NTS on day 14. Representative pictures are shown. Magnification x200 (HE), x400 (CD41^+^) and x600 (Ter119^+^).

**Fig 8:**
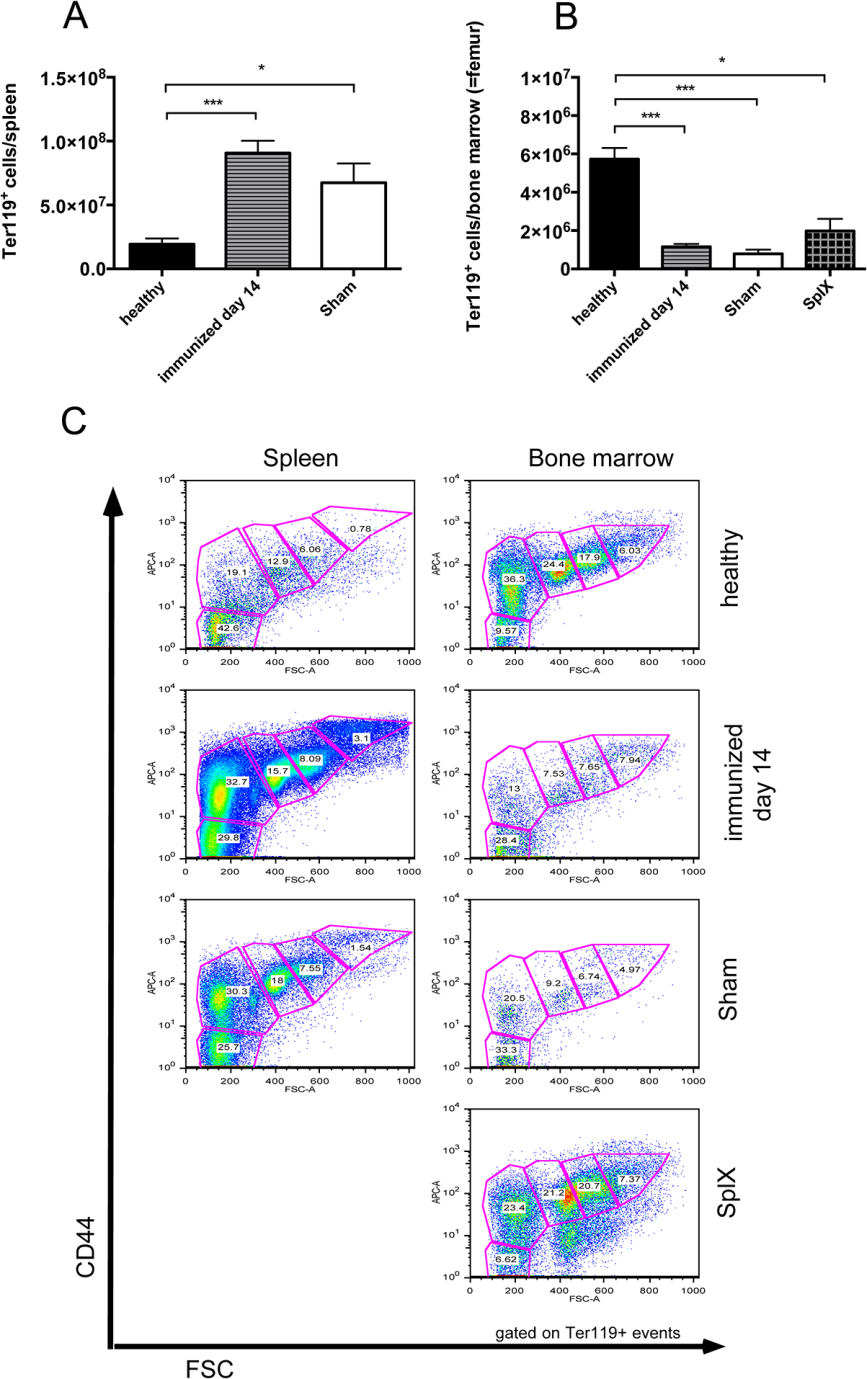
NTS and immunisation increase erythroid progenitor cells in the spleen and repress the bone marrow haematopoiesis. Spleens (A, C) and bone marrow (B, C) of healthy (black bars, n = 5) and immunized (lined bars, n = 5) mice on day 14 as well as mice that either underwent splenectomy (SplX, squared bar, n = 5) or sham-operation (Sham, white bars, n = 5) prior to the induction of NTS were evaluated for erythroid precursors in quantitative flow cytometry on day 14. (A, B) Data are given as means ± SEM. (C) Representative dot plots are shown. *p<0.05, ***p<0.001.

Since we found increased spleen weight during the course of NTS, we evaluated different splenic cell populations of nephritic animals. Interestingly, when gated on all leukocytes, relative numbers of CD4^+^ and activated CD4^+^CD69^+^ T cells (Fig 5A and S7 Table), CD8^+^ and activated CD8^+^CD69^+^ T cells (Fig 5C and S7 Table) as well as CD11b^+^SSC^low^ cells, reflecting monocytes, (Fig 5E and S7 Table) significantly decreased in the spleen of immunized mice and mice 14 days after NTS induction as compared to healthy controls. Only the CD11b^+^SSC^hi^ population, accounting mainly for neutrophil granulocytes, increased in spleens of immunized mice and mice 14 days after NTS as compared to healthy controls in relative and absolute analysis (Fig 5E, 5F and S7 Table). CD4^+^ and CD8^+^ T cells also decreased in absolute numbers in spleens of immunized and nephritic mice on day 14, although this decrease did not reach statistical significance. Interestingly, absolute numbers showed a significant increase of CD11b^+^SSC^low^ monocytes in spleens of immunized mice as compared to healthy and nephritic mice 14 days after NTS induction. Of note, also relative amounts of B220+ B cells remained stable in their relative numbers in the spleens of NTS mice (data not shown). Since the increase in spleen weight was not due to increased leukocytes, we evaluated the area of the red pulp in spleens of healthy mice, immunized mice, and mice 14 days after induction of NTS by staining for the macrophage marker F4/80 since this marker is highly expressed by red pulp macrophages, whereas monocytes and monocyte-derived macrophages are F4/80low [16]. Strikingly, the red pulp significantly increased in mice 14 days after NTS whereas the white pulp decreased (Fig 6 and S8 Table). Also, the red pulp of immunized mice increased when compared to healthy mice (Fig 6 and S8 Table), however did not reach statistical significance. In HE stainings we detected giant cells in immunized mice and mice 14 days after NTS induction (Fig 7), which were CD41^+^—indicative for megakaryocytes and platelets (Fig 7). Furthermore, we evaluated the erythrocyte lineage by staining for Ter119 and found a strikingly different staining pattern indicating a shift towards blastic Ter119^+^ cells 14 days after immunisation only and NTS induction (Fig 7). To further characterize this lineage we performed flow cytometry for Ter119 as well as CD44. We found a significant increase in proerythroblasts and reticulocytes in spleens 14 days after NTS induction (Fig 8A and S9 Table). In parallel, the erythroid lineage decreased significantly in the bone marrow of mice 14 days after NTS induction as compared to healthy controls (Fig 8B and S9 Table). These inverse changes of the erythroid lineage in the spleen and bone marrow were also observed when mice were only immunized with rabbit IgG, but received no GBM antiserum (Fig 8A and 8B), even though they had haemoglobin levels comparable to healthy mice (Fig 4). In splenectomized mice bone marrow erythropoiesis was increased compared to sham-operated mice 14 days after NTS induction, but significance was not reached (Fig 8).

### NTS and immunization only lead to a systemic inflammatory state

**Fig 9:**
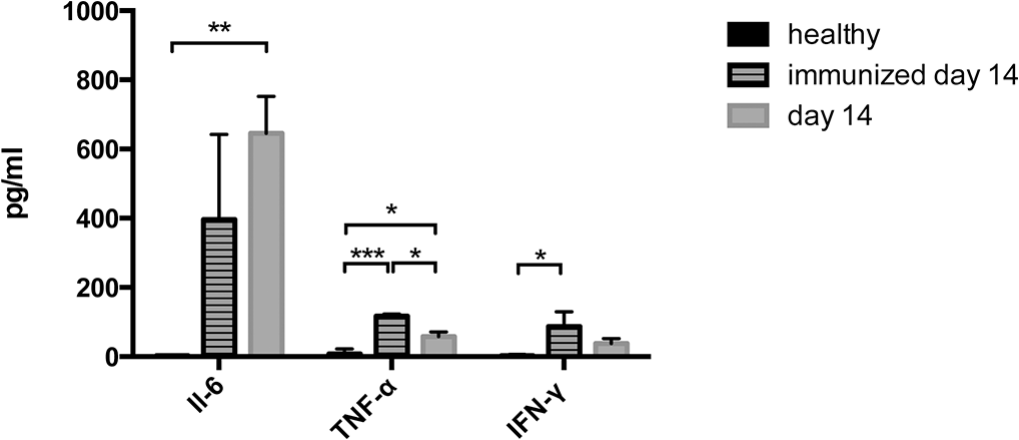
Immunized and nephritic mice display increased levels of serum, Il-6, TNF-α and IFN-γ. Serum Il-6, TNF-α and IFN-γ were measured in healthy (n = 4), immunized mice on day 14 (n = 4) and nephritic mice on day 14 (n = 4). Data are given as means ± SEM. *p<0.05; **p<0.01; ***p<0.001.

Systemic inflammation is one major driving force of extramedullary haematopoiesis [17]. To evaluate whether our mice display a systemic inflammatory state, serum-levels for Il-6, TNF-α and IFN-γ were performed. We found significantly increased Il-6 and TNF-α serum levels in nephritic mice on day 14 (Fig 9 and S10 Table). IFN-γ levels also increased in nephritic mice, but significance was not reached. Also, immunized mice displayed increased serum-levels of respective pro-inflammatory cytokines (Fig 9 and S10 Table).

### Migration of erythroid precursors to the spleen in NTS seems to be partly dependent on the Cxcr4/Cxcl12 axis

**Fig 10:**
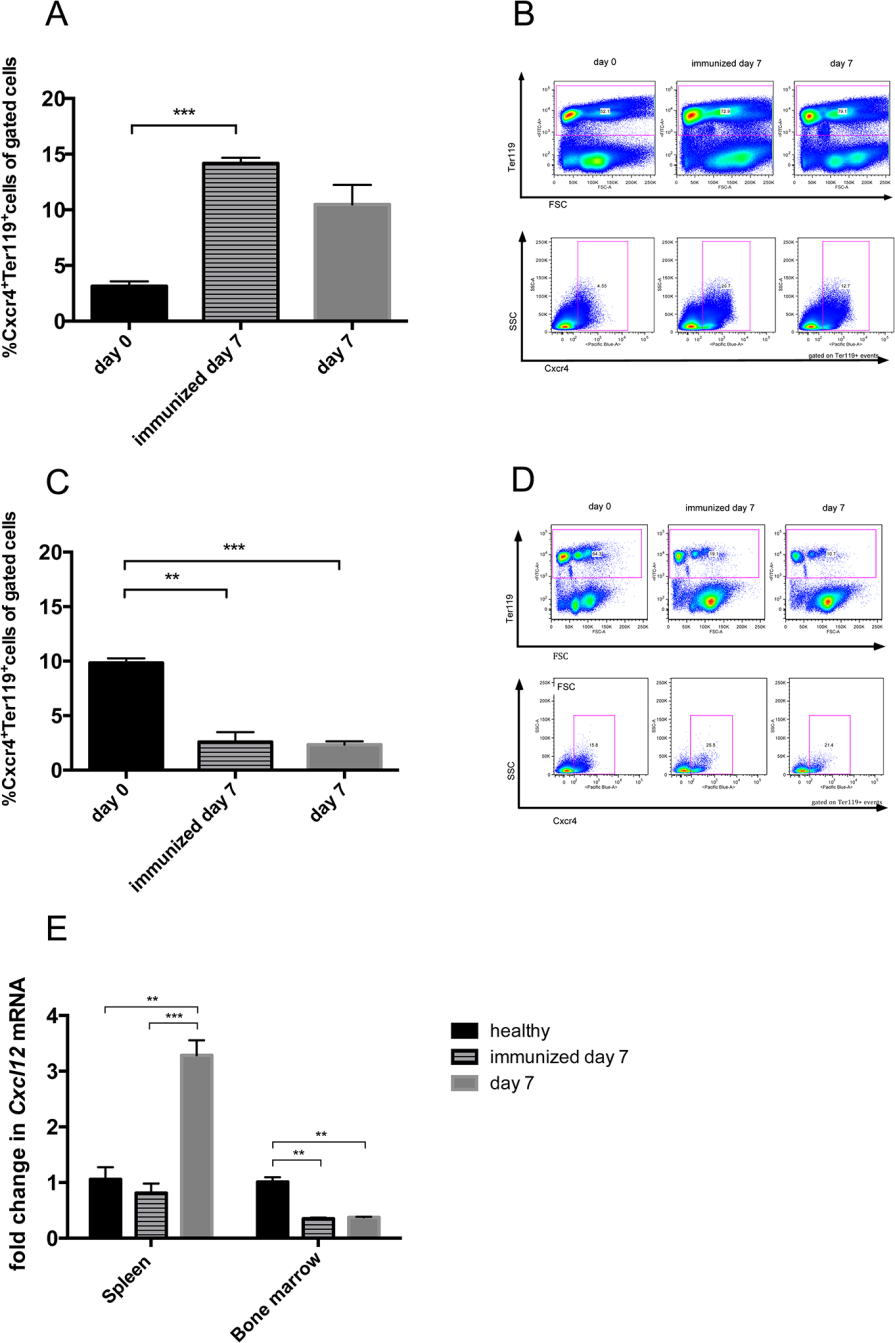
Cxcr4 expressing erythroid progenitors seem to migrate from the bone marrow to the spleen following a Cxcl12 gradient in immunized and nephritic mice. Spleens (A, B) and bone marrow (C, D,) of healthy (black bars, n = 3), immunized (lined bars, n = 4) and nephritic mice on day 7 (grey bars, n = 4) were evaluated for Ter119^+^Cxcr4^+^ cells by performing relative flow cytometry. The expression of *Cxcl12* mRNA was evaluated by quantitative real-time PCR of total RNA from spleens and bone marrow of healthy, immunized and nephritic mice on day 7 (E). Data are given as means ± SEM. *p<0.05; **p<0.01; ***p<0.001.

To evaluate whether the development of extramedullary haematopoiesis in NTS is dependent on chemokine-mediated mechanisms, we examined the expression patterns of chemokines and their respective receptors known to be involved in the migration of erythroid precursor cells. Indeed, we found that erythroid precursors in the spleen were predominantly Cxcr4^+^ (Fig 10A and 10B and S11 Table), while bone marrow resting Ter119^+^ cells were mainly Cxcr4^-^ (Fig 10C and 10D and S11 Table) 7 days after induction of NTS or immunization only. In line, we found that *Cxcl12* mRNA, a chemokine important for hematopoietic stem/progenitor cell homeostasis regulation [18], increased in the spleen on day 7 of NTS, while it decreased in the bone marrow of immunized and nephritic mice (Fig 10E and S12 Table). Of note, we did not observe significant mRNA changes in *Ccl2* and *Ccl7*, which were previously described to be involved in the development of extramedullary haematopoiesis [19], in the spleen during NTS (data not shown).

## Discussion

Secondary lymphoid organs such as the spleen and lymph nodes have been proven to play important roles in immune activation as well as regulation in autoimmune diseases such as immune-complex GN [1,2,15]. Nevertheless, the distinct role of the spleen in the pathogenesis of GN is unclear so far. Here, we provide evidence that the spleen does not play a key role in the pathogenesis of *NTS per se*, but partly compensates concomitant anaemia due to extramedullary haematopoiesis (EMH).

The spleen is important in activation and regulation of cellular immune responses, but also in production and resolution of antibodies and immune-complexes, respectively. In the experimental model of NTS, which is close to human rapid-progressive immune-complex GN, it has been shown that immune regulation processes such as homing of regulatory T cells via C-C chemokine receptor 7 takes mainly place in the regional draining lymph nodes, but also in the spleen [1,2]. To distinguish the roles of lymph nodes and spleen in the pathogenesis of NTS, we performed splenectomy followed by NTS induction and followed the mice for a maximum of 28 days. Interestingly, splenectomy had neither an effect on the renal phenotype, nor on the autologous and heterologous antibody levels in the serum and their deposition on the GBM during the observation period. This indicates that B cell activation and maturation, but also T cell activation and regulation in NTS takes place in the lymph node rather than in the spleen. In humans, data on splenectomy and the development of autoimmune diseases and GN are scarce. Few case reports showed either protective or harmful effects of splenectomy on the course of different forms of GN [8–10].

Nevertheless, the size of the spleen significantly increased during the course of NTS, but this enlargement was due to an increase in the red pulp rather than the white pulp. In line, signs of EMH such as megakaryocytes and proerythroblasts were detected in the spleen of mice subjected to NTS. In parallel, we detected a repressed erythropoiesis in the bone marrow. Recently, EMH has been described in a model of systemic lupus erythematosus [20,21]., but to our knowledge this is the first description in the NTS model. Although a complete understanding of the mechanisms underlying EMH is not fully elucidated, 4 major theories encompass most of the pathophysiologic causes: (1) bone marrow failure; (2) bone marrow stimulation; (3) tissue inflammation, injury, and repair; and (4) abnormal systemic or local chemokine production [17]. In the model of NTS, EMH is probably mainly due to systemic inflammation as well as local chemokine production. The systemic inflammation is reflected by increased Il-6, TNF-α and IFN-γ serum-levels in mice 14 days after NTS induction. Belyaev and coworkers recently described systemic IFN-γ to trigger the secretion of the chemokines Ccl2 and Ccl7 thereby leading to splenic EMH in a Ccr2-dependent manner in acute malaria [19]. However, we did not detect changes of *Ccl2* and *Ccl7* mRNA in the spleen during NTS. Another recent publication detected EMH and decreased bone marrow haematopoiesis comparable to our observations in a murine model of lupus nephritis. This study found EMH to be dependent on toll like receptor 7 and Cxcl12 [21]. In line, EMH in NTS seems to develop partly via the migration of Cxcr4 positive erythroid precursor cells along a Cxcl12 gradient from the bone marrow to the spleen. Interestingly, only one leukocyte subpopulation, namely CD11b^high^ cells, increased in the spleen of nephritic mice. This fact additionally supports our evidence on the importance of the Cxcr4/Cxcl12 axis in the NTS model, since neutrophil migration was previously found to depend on the Cxcr4/Cxcl12 axis [22]. Nevertheless, there are probably also other chemokines involved in the pathogenesis of NTS, which have not been unravelled so far. Additional experiments with respective knock-out mice or antibody-blockade are needed to prove the significance of the Cxcr4/Cxcl12 axis in the pathogenesis of EMH in NTS.

Interestingly, EMH also took place in mice that were only immunized without the injection of the anti-GBM antiserum even though these mice did not develop anaemia. According to our results, immunization also induced systemic inflammation reflected by increased IFN-γ levels and changes in the Cxcr4^+^ erythroid precursor cell content in the spleen and bone marrow. *CxCl12* mRNA accordingly decreased in the bone marrow of immunized only mice, but surprisingly we did not detect an increase in *CxCl12* mRNA in the spleen. We thus speculate that the crucial function of Cxcl12 is the releasing effect of a Cxcl12 decline on progenitor cells in the bone marrow rather than an increase e.g. in the spleen as shown previously by others [23]. These changes support the theory that systemic inflammation is the cause of EMH rather than anaemia due to renal failure. Notably, we did not see an increase in the *Cxcl12* mRNA in the spleen of immunized mice possibly reflecting a stimulus other than systemic inflammation for Cxcl12 production. Again, additional experiments are needed to clarify this issue.

In our hands, EMH in the spleen was able to partly compensate reduced bone marrow haematopoiesis since splenectomized mice displayed decreased haemoglobin levels.

The implications for the human situation in GN remain unclear and have not been studied so far. Further research is needed to evaluate spleen EMH in patients with rapid-progressive GN.

Taken together, we here provide evidence that the spleen does not play a key role in the pathogenesis of NTS. Concomitant anaemia develops in NTS and is accompanied by EMH in the spleen and repression of bone marrow haematopoiesis. Chronic systemic inflammation as reflected by increased IFN-γ levels accompanies EMH and might contribute to the migration of Cxcr4 positive erythroid precursor cells from the bone marrow to the spleen. In NTS, EMH was found to partly compensate anaemia.

## Supporting Information

S1 Table Spleenweight (Fig 1A). (XLSX)

S2 Table Albuminuria (Fig 2A). (XLSX)

S3 Table PAS (Fig 2B). (XLSX)

S4 Table Immunohistochemistry (Fig 2E and 2F). (XLSX)

S5 Table IgG (Fig 3A and 3B). (XLSX)

S6 Table Haemoglobin (Fig 4). (XLSX)

S7 Table FC_Spleen (Fig 5). (XLSX)

S8 Table F4/80 (Fig 6B). (XLSX)

S9 Table FC_Ter119 (Fig 8AB). (XLSX)

S10 Table Cytokines (Fig 9). (XLSX)

S11 Table FC_Ter119_CXCR4 (Fig 10). (XLSX)

S12 Table Cxcl12 (Fig 10E). (XLSX)
